# Re-examination of the impact of some non-pharmaceutical interventions and media coverage on the COVID-19 outbreak in Wuhan^☆^

**DOI:** 10.1016/j.idm.2021.07.001

**Published:** 2021-07-17

**Authors:** Ao Li, Yang Wang, Pingping Cong, Xingfu Zou

**Affiliations:** a Department of Applied Mathematics, University of Western Ontario London, Ontario, N6A 5B7, Canada; b School of Mathematics and Statistics, Northeast Normal University, 5268 Renmin Street, Changchun, Jilin, 130024, PR China

**Keywords:** COVID-19, SARS-CoV-2, Kermack-McKendrick SIR model, Non-pharmaceutical intervention

## Abstract

In this paper, based on the classic Kermack-McKendrick SIR model, we propose an ordinary differential equation model to re-examine the COVID-19 epidemics in Wuhan where this disease initially broke out. The focus is on the impact of all those major non-pharmaceutical interventions (NPIs) implemented by the local public healthy authorities and government during the epidemics. We use the data publicly available and the nonlinear least-squares solver *lsqnonlin* built in MATLAB to estimate the model parameters. Then we explore the impact of those NPIs, particularly the timings of these interventions, on the epidemics. The results can help people review the responses to the outbreak of the COVID-19 in Wuhan, while the proposed model also offers a framework for studying epidemics of COVID-19 and/or other similar diseases in other places, and accordingly helping people better prepare for possible future outbreaks of similar diseases.

## 1 Introduction

Coronaviruses are medium-sized RNA viruses with the crown-like appearance of the surface projections (Kahn & McIntosh, 2005; Tyrrell et al., 1975). They can infect the respiratory, gastrointestinal, hepatic and central nervous system of human, livestock, birds and bats *etc.* as their important pathogens (Chen et al., 2020; Chen & Guo, 2016; Ge et al., 2013; Wang et al., 2006). Coronaviruses were first discovered in 1965 from the respiratory tract of an adult with a common cold and were later officially accepted as a new genus of viruses (Kahn & McIntosh, 2005). They had not caused large-scale outbreaks until the emergence of the Severe Acute Respiratory Syndrome (SARS) from southern China in 2002 (Hui & Zumla, 2019; World Health Organization, 2020b). In 2012, a new lethal disease caused by a coronavirus, Middle East Respiratory Syndrome (MERS), outbroke in Saudi Arabia. Shortly after, the MERS also had an outbreak in South Korea in 2015 (World Health Organization, 2020a; Zumla et al., 2015).

More recently, a novel coronavirus disease broke out again. On 21 December 2019, a number of unknown pneumonia cases occurred in Wuhan, China (Tan et al., 2020). On 7 January 2020, a novel coronavirus was discovered from one patient. The novel coronavirus was firstly named as 2019 novel coronavirus (2019-nCoV) by the World Health Organization (WHO) on 10 January 2020, and later was renamed as Severe Acute Respiratory Syndrome Coronavirus 2 (SARS-CoV-2 in short), and the disease it causes has been officially named as coronavirus disease of 2019 (COVID-19 in short) (Tan et al., 2020; World Health Organization, 2019). Then the epidemic of COVID-19 rapidly grew firstly in Wuhan and soon spread to many other places in China with worrisome speed. As of 30 January 2020, there were 9, 692 confirmed cases and 213 death cases reported in mainland China, distributed in all 31 provinces. Hong Kong, Taiwan, Macao and many countries soon started to report confirmed cases. On 30 January 2020, the novel coronavirus outbreak was declared as a “public health emergency of international concern” (PHEIC) by the International Health Regulations Emergency Committee of the World Health Organization. Later, SARS-CoV-2 has been spread to more and more countries, leading to a pandemic, which has reported over 67 millions of infections and caused more than 1.5 million deaths around the world as of 9 December 2020 (World Health Organization, 2020c). In addition to loss of lives, there is a huge negative impact on the global economy.

For this disastrous disease, efforts have been and are being made in locating the source of this coronavirus, finding various treatments and developing effective vaccines. On the other hand, re-examining why and how this virus has been spread to the globe is important and worth doing, because such a re-examination will help human beings better prepare for possible future outbreaks of other similar infectious diseases, particularly diseases caused by new coronaviruses. For this purpose, it seems to be natural and reasonable to start with a re-examination of controls and interventions in Wuhan, the city where SARS-CoV-2 was firstly reported in late 2019 and COVID-19 firstly outbroke in late 2019 and early 2020. Because this is a newly emerged disease, there was no known and effective medical treatment when it broke up in Wunan, neither there was a vaccine (almost one year after the outbreak, reliable vaccines were developed and gradually became avaialble), we will focus on *non-pharmaceutical interventions (NPIs) and media coverage*. To this end, we first briefly review the four key measures implemented in Wuhan and their timings, which we believe played crucial roles.

(M1) *Closure of HSWM.* Once realizing that the majority of the earliest cases had exposure to a food market called Huanan seafood wholesale market (HSWM), it was suspected that the market might be the source of infection. Then on 1 January 2020, the market was closed and disinfected to block the transmission. At that time, it was believed that the pathogen was solely from that market and was the only source of infection, and there was no human-to-human transmission.
(M2) *Announcement of human-to-human transmission.* Since the closure and hygienization of HSWM, a continued and even faster increase in the number of reported COVID-19 cases seemed to suggest that human-to-human transmission of the coronavirus was also possible. This possibility was soon confirmed and officially announced to the public on 20 January 2020 through various media. This announcement has a crucial impact, because before this, without realizing human-to-human transmission, many large scale activities and gatherings had been going on as scheduled, and traditional pre-Chinese-New-Year shoppings were as active as in all previous years, with participants and shoppers having no or little personable protections.
(M3) *Lockdown of the City.* Further realizing the severity of the epidemic of COVID-19, three days after announcing human-to-human transmission, the government announced lockdown of the whole city of Wuhan starting on 23 January 2020, and the lockdown was in the most serious sense that all businesses were shut down, all public transportations including buses, subways and ferry lines were suspended within Wuhan; additionally, all airports and train stations were halted. Shortly after that, other cities in Hubei province also announced strict restrictions similar to Wuhan.
(M4) *Making available extra medical resources.* Realizing the big shortage in medical facilities in treating COVID-19 patients, on 23 January 2020, Wuhan government also decided to build a special emergency hospital specifically designed for treating COVID-19 patients, named Huoshenshan Hospital. The construction was completed in 10 days and on 4 February 2020, it started admitting the first group of patients. Huoshenshan Hospital was equipped with 1, 000 beds, 30 intensive care units and several quarantine wards. A few days later, a second special emergency hospital, Leishenshan Hospital, was built and opened on 8 February 2020. In addition, since 14 February 2020, 16 mobile cabin hospitals, called Fangcang Hospitals in Chinese, were constructed which could provide a total of more than 20, 000 beds. Together with the addition of these special hospitals, tens of thousands of front medical workers (doctors and nurses), recruited from other provinces of China, arrived in Wuhan group by group, and worked in all hospitals 24 hours a day and 7 days a week. These added new facilities and health workers greatly improved the capability of rapid diagnosis, efficient isolation and timely treatments.

With the above unprecedented measures, plus some other efforts, China had successfully controlled the COVID-19 outbreak. The National Health Commission of China declared that “domestic transmission had almost been blocked” on 31 March 2020. Wuhan, China's coronavirus epicentre, ended 76-day lockdown on 8 April 2020. The numbers of new infections and active cases reported in China maintain at a very low level since then until today.

We point out that mathematical modelling can play a key role in providing evidence-based information for health policy decision makers and it has been recognized by the World Health Organization (Tang et al., 2020). Some mathematical models have been established to quantify the effectiveness of some NPIs. Particularly, some recent studies have shown that those strategies based on mathematical modelling can effectively prevent the spread of SARS-CoV-2. For example, (Tang et al., 2020) used a deterministic compartmental (SEIR) model, and through estimating the effective daily reproduction ratio and sensitivity analyses, they showed that the interventions such as intensive contact tracing followed by quarantine and isolation can effectively reduce the risk of transmission. (Shen et al., 2020) proposed a mathematical model to analyze how the lockdown measure impacts the spread of SARS-CoV-2 (which was named as 2019-nCov during the earlier stage of the outbreak) by investigating various scenarios like delayed implementation and no lockdown. Their results show that lockdown can effectively reduce new infections and deaths. (Hellewell et al., 2020) designed a stochastic transmission model to quantify the potential effectiveness of contact tracing and isolation of cases at controlling the novel coronavirus disease. Without other controls, case isolation, contact tracing and blocking transmission from infected people would be sufficient to prevent a new COVID-19 outbreak. (Yang et al., 2020) built an SEIR model to derive the epidemic curve, in order to analyze the impact of these control measures (such as large-scale quarantine, strict travel restriction and extensive monitoring of suspected cases) on the epidemic. They predicted that if the implementation of these measures was delayed by five days, the size of the outbreak in mainland China would triple. (Cui et al., 2020) also explore the effect of quarantine on COVI-19 dynamics in China. (Lai et al., 2020) developed a modelling framework that uses daily travel networks to simulate different outbreak and intervention scenario across China, using a questionable simple SEIR model for each patch. In addition to these interventions, media coverages have made a great contribution to fighting COVID-19 epidemics. After the novel coronavirus was firstly reported by Wuhan Municipal Health Commission on 11 January 2020, the National Health Commission of China updates the epidemic report every day. Information spread out immediately via media and quickly attracted the attention of public. Real-time data, intensive epidemic news and disease prevention knowledge can be easily found on many popular network platforms and commonly used mobile applications. People's awareness and fear of the outbreak in turn influence individuals' behaviours, which may lead to strengthened self-protection such as staying home as much as possible, wearing face masks in public places and washing hands frequently (Funk et al., 2009). There have been some works on modelling the role of media coverage in controlling the COVID-19 in China which quantitatively and qualitatively reveal the effects; see, e.g. (Chang et al., 2020; Liu et al., 2021; Zhou et al., 2020), and the references therein.

In this paper, we focus on the coronavirus outbreak in Wuhan, where the first case was reported and had the majority of infections and medical resources. Based on the classic Kermack-McKendrick SIR model, we propose an SEIR model that incorporates some important factors related to the four key measures described in (M1)-(M4), including the impact of media coverage and official announcements. The model is a system of ordinary differential equations with *time-dependent parameters*, reflecting the timings of implementing those measures. We then use the data available to us for the outbreak in Wuhan to validate some basic model parameters. Finally, we will vary some non-biological parameters that represent various aspects of certain decisions and control measures by the governments and public health authorities, particularly the values of those critical time points (dates) when those related important measures were taken/implemented, to see if there would have been significant changes in epidemics. This would help us to review Wuhan's responses to the COVID-19 outbreak and seek improvement for future when facing similar situations of disease outbreaks.

The rest of the paper is organized as follows. In Section 2, we give detailed descriptions on the model; we also activate the model estimating the model parameters: some are directly obtained from published research works while the others are obtained using the data available and the nonlinear least-squares solver *lsqnonlin* that is built in MATLAB. In Section 3, with the activated model, we explore the impact of the timings for the four major control measures. In Section 4, we summarize main results of the paper and their implications; we also discuss some limitations of our model and possible improvements.

## 2 Mathematical model

We begin by recalling the classic and simple Kermack-McKendrick SIR ordinary differential equation model for the transmission dynamics of an infectious disease:

(1) $$\left\{\begin{array}{l} \frac{dS}{dt}=-\beta SI,\\ \frac{dI}{dt}=\beta SI-\left(\alpha +\gamma \right)I,\\ \frac{dR}{dt}=\left(\alpha +\gamma \right)I. \end{array}\right)$$

Here the population is divided into three classes with *S*(*t*), *I*(*t*) and *R*(*t*) denoting the subpopulations of these classes: susceptible, infectious and removed classes. The parameter *β* is the transmission rate. An SIR type model assumes that recovered individuals will carry immunity for the disease. Moreover, the removed class includes those died of the disease and those recovered from the disease. Here in (1), for the convenience of data fitting later, we distinguish these two sub-classes by incorporating two different parameters *α* and *γ*, with the former denoting the recovery rate and the latter being the disease caused death rate. In (1), the demography of the host population is omitted/ignored. This is reasonable for those diseases that emerge fast and the durations of such diseases are much shorter compared with the life span of host population.

Now we expand (1) to a model for the COVID-19 outbreak in Wuhan that had the featured NPIs described in (M1)-(M4). Firstly, noting that the time from exposure to symptom onset of COVID-19 is thought to extend to 14 days and presymptomatic patients are possible to be contagious, we need to add a compartment for the population during the *incubation period*, denoting it by *E*(*t*). We point out that *E*(*t*) here is not the population in the *latent period* like in many other SEIR type model, and when it comes to modelling, the susceptible individuals may be infected by contact with *E*(*t*) or *I*(*t*). Secondly, since the source of the novel coronavirus is speculated to be the wildlife traded in Huanan seafood wholesale market and early infections were believed to mainly from this market, possibly through the environment-to-human transmission, we need another compartment for the concentration of SARS-CoV-2 in the environment, denoting it by *V*(*t*). With these two variables incorporated together with the above justifications, we then naturally expand the Kermack-McKendrick model (1) into the following model of ordinary differential equations

(2) $$\left\{\begin{array}{l} \frac{dS}{dt}=-\beta_{10}\left(p_{1}S\right)V-\beta_{20}\left(p_{2}S\right)I-\beta_{30}\left(p_{3}S\right)E,\\ \frac{dE}{dt}=\beta_{10}\left(p_{1}S\right)V+\beta_{20}\left(p_{2}S\right)I+\beta_{30}\left(p_{3}S\right)E-\sigma E,\\ \frac{dI}{dt}=\sigma E-\alpha I-\gamma I,\\ \frac{dR}{dt}=\alpha I+\gamma I,\\ \frac{dV}{dt}=\mu -dV. \end{array}\right)$$

Here the variables *S*, *E*, *I*, *R* and *V* have been explained above, and all the parameters are non-negative with their biological meanings explained below:

(i) *p*_1_ is the fraction of susceptible population that visit Huanan seafood wholesale market; *p*_2_ and *p*_3_ are the fractions of the susceptible population that have a chance to be exposed to *I* class and *E* class individuals respectively (see more discussion later);
(ii) *β*_10_, *β*_20_ and *β*_30_ are *V*(*t*)-to-*S*(*t*), *I*(*t*)-to-*S*(*t*) and *E*(*t*)-to-*S*(*t*) transmission rates, respectively;
(iii) *σ* is the rate at which exposed individuals develop symptoms (incubation period ends);
(iv) *α* is the recovery rate;
(v) *γ* is disease-induced death rate;
(vi) *μ* is the discharging rate of the novel coronavirus concentration by infected animals in Huanan seafood wholesale market;
(vii) *d* is the natural mortality rate of SARS-CoV-2 when exposed in the environment.

As mentioned in the introduction, the NPIs and media coverage played an important role in controlling the epidemics, and such information and factors should not be neglected when modelling the epidemics. Such interventions will not change the values of those biological parameters such as infection rate, death rate and recovery rate *etc*. However, these factors will alter the values of some non-biological parameters in the model at certain time points during the epidemic. We will elaborate in details below.

Firstly, we realize that the essence and the effect of all NPIs lie in reducing the *susceptible population available for infection*, letting us call it *available susceptible population* which differs from the total susceptible population. In our model (2), such effects are reflected by the three fractions *p*_*i*_, *i* = 1, 2, 3. Starting with the environment-to-human transmission channel, we assume that environment-to-human transmission only occurred in the beginning of the outbreak in Huanan seafood wholesale market. After the closure of the market on 1 January 2020, such transmission is prevented and *V*(*t*) vanishes. This can be reflected by the switching nature of parameter *p*_1_:

(3) $$p_{1}=p_{1}\left(t\right)=\left\{\begin{array}{ll} p_{10}, & t\in \left[t_{0},T\right),\\ 0, & t⩾T. \end{array}\right)$$

Next, by the meanings of *p*_2_ and *p*_3_, it is seen that *p*_2_*S* and *p*_3_*S* account for the numbers of susceptible individuals that are *available for infection* by *I* class and *E* class individuals respectively. Let us call *p*_2_*S* the number of *I*-available susceptibles and *p*_3_*S* the number of *E*-available susceptibles. Obviously, *p*_2_ and *p*_3_ are significantly impacted by the various restrictions and control measures set by the government, as well as the information from various platforms of public health organizations and media. To reflect these impacts, we propose the following forms for the two fractions *p*_2_ and *p*_3_:

(4) $$\begin{array}{cc} p_{2}\left(t\right) & =\frac{1}{1+m_{1}\left(t\right)I\left(t\right)+m_{2}\left(t\right)\max\left\{0,\Delta I\left(t\right)\right\}}\cdot \frac{1}{1+m_{3}\left(t\right)}\cdot \frac{1}{1+m_{4}\left(t\right)}\\ p_{3}\left(t\right) & =\frac{1}{1+m_{1}\left(t\right)I\left(t\right)+m_{2}\left(t\right)\max\left\{0,\Delta I\left(t\right)\right\}}\cdot \frac{1}{1+m_{3}\left(t\right)}\cdot \frac{1}{1+m_{5}\left(t\right)} \end{array}$$

Here, we have assumed that not only the number of infectious individuals *I*(*t*) but also its increment Δ*I*(*t*) = *I*(*t*) − *I*(*t* − 1) will affect people's behaviours and activities, as the increment suggests a trend of the epidemics in certain sense. The coefficients *m*_1_(*t*) and *m*_2_(*t*) represent the level of media coverage, corresponding to the situation mentioned in (M2). We point out that max{0, Δ*I*(*t*)} is a technical modification of Δ*I*(*t*) to avoid negativity and will be denoted by $\tilde{\Delta }I\left(t\right)=\max\left\{0,\Delta I\left(t\right)\right\}$ subsequently. Also, the lockdown described in (M3) means that more people were forced to stay home (due to restriction rules, as well as lack of public transportation and closure of commercial stores and restaurants, *etc.*), and this would surely reduce the fractions of the *I*-available and *E*-available susceptible classes. Thus, we incorporate the quantity *m*_3_(*t*) to reflect such an effect.

Corresponding to (M4), the main effects of the addition of those extra medical and health resources are twofold. Firstly, more hospital rooms and more doctors and nurses meant broader and more intensive treatments for diagnosed infected patients leading to a higher recovery rate and a lower disease-induced death rate. We reflect this by assuming that parameters *α* and *γ* are step functions, with *α* = *α*(*t*) jumping from a lower level to a higher one while *γ* = *γ*(*t*) switching from a higher level to a lower one, after such resources being available. Such an assumption is supported by the related data available for the public. Secondly, these extra resources also increased the capability in extensive and intensive contact tracing, large scale testing and timely quarantine; and all these effectively reduced the contact between individuals in *I* and *E* groups with people in *S* group. We introduce *m*_4_(*t*) and *m*_5_(*t*) in the forms of *p*_2_(*t*) and *p*_3_(*t*) given by (4) to account for the impacts.

**Fig. 1:**
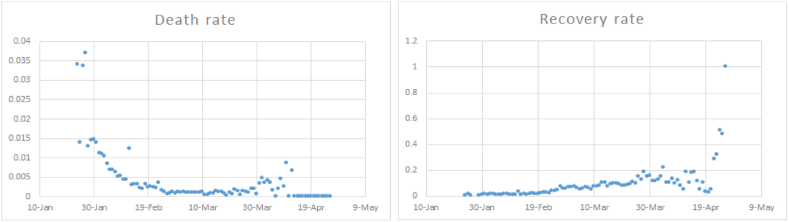
The daily disease-induced death rate and recovery rate.

Wuhan Municipal Health Commission reports the outbreak everyday since 23 January 2020 on their official website(Wuhan Municipal Health Co, 2020), including the number of daily new cases, daily new deaths, daily new recovered, total confirmed, total deaths and total recovered in Wuhan. We can easily obtain the number of active cases from the data, and use the ratios daily new deaths/active cases and daily new recovered/active cases to approximate daily disease-induced death rate and recovery rate, respectively, as plotted in Fig. 1. The active cases in Wuhan firstly became zero on 26 April 2020 and no more new cases appeared until 9 May 2020. After then, the number of active infections remains under a very small value, and many of them are imported cases. Hence, we only collect the data between 23 January 2020 and 26 April 2020. Until 26 April 2020, there were 50, 333 people in Wuhan confirmed to be infected by the novel coronavirus, among whom 3, 869 (7.69*%*) died due to the disease and 46, 4646 (92.31*%*) recovered. Fig. 1 implies that it is not reasonable to use constant values for disease-induced death rate and recovery rate in the model to describe the entire outbreak. Ignoring the points near the starting and ending dates which are abnormal due to the small amount of active cases, the daily death rate is relatively high in the beginning and then it decreases to a low level, while the recovery rate is very low at first and then it keeps increasing. This is because two special emergency hospitals and many mobile cabin hospitals became available and many medical personnel from all other provinces were later recruited to Wuhan to help, as mentioned in (M4). To make it simple, we set 16 February 2020 as a turning point and use average values to represent the rates for each period.

**Fig. 2:**
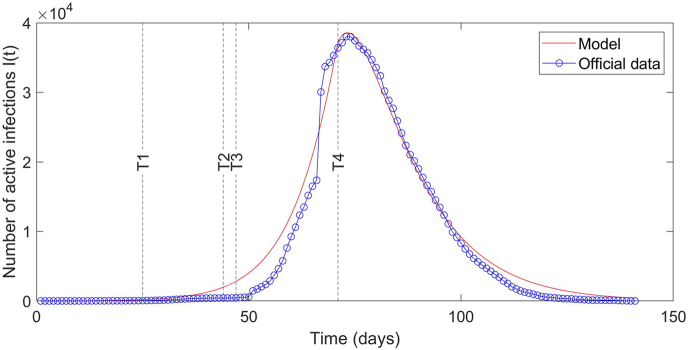
The numerical simulation of COVID-19 outbreak in Wuhan with parameter values given in Table 1, Table 2 and initial data [*S*(*t*_0_), *E*(*t*_0_), *I*(*t*_0_), *V*(*t*_0_)] = [11081000, 2, 1, 200], compared with the official data released by Wuhan Municipal Health Commission.

Now we rewrite (2) as the following more standard form

(5) $$\left\{\begin{array}{l} \frac{dS}{dt}=-\beta_{1}\left(t\right)SV-\beta_{2}\left(t\right)SI-\beta_{3}\left(t\right)SE,\\ \frac{dE}{dt}=\beta_{1}\left(t\right)SV+\beta_{2}\left(t\right)SI+\beta_{3}\left(t\right)SE-\sigma E,\\ \frac{dI}{dt}=\sigma E-\alpha \left(t\right)I-\gamma \left(t\right)I,\\ \frac{dR}{dt}=\alpha \left(t\right)I+\gamma \left(t\right)I,\\ \frac{dV}{dt}=\mu -dV, \end{array}\right)$$

where *β*_*i*_(*t*) = *β*_*i*0_*p*_*i*_(*t*), *i* = 1, 2, 3.

Notice that there are four critical dates which played crucial roles in controlling the epidemic in Wuhan. These four dates divide the duration of the epidemic into five stages. The article (Huang et al., 2020) published in The Lancet indicates that the first patient started experiencing symptoms on 1 December 2019, but the symptom onset date in official data is 8 December 2019 which is also used in the paper (Li et al., 2020). Here, we adopt 8 December 2019 as the initial time (*t* = 1), and the aforementioned four dates are accordingly translated into the corresponding time points *t*_1_, *t*_2_, *t*_3_ and *t*_4_ as described in Table 1. The parameter *p*_1_ switches to zero from *t*_1_ because of the closure of Huanan seafood wholesale market. Human-to-human transmission announced on *t*_2_ affects *m*_1_ and *m*_2_. The lockdown of Wuhan city alters *m*_3_ from *t*_3_. At and after *t*_4_, the values of parameters *α*, *γ*, *m*_4_ and *m*_5_ are changed to different constants in response to the availability of extra medical resources.

**Table 1:** Critical time points in the outbreak of COVID-19 in Wuhan.

Time (day)	Date	Actions or events
*t*_0_ = 1	Dec. 8, 2019	The first patient identified developed symptoms.
*t*_1_ = 25	Jan. 1, 2020	Huanan seafood wholesale market was closed.
		Human-to-human transmission announced officially
*t*_2_ = 44	Jan. 20, 2020	and publicly on media; people started to take
		personal protection measures.
*t*_3_ = 47	Jan. 23, 2020	Wuhan was locked down.
*t*_4_ = 71	Feb. 16, 2020	Extra medical resources were available.
*t*_5_ = 141	Apr. 26, 2020	Active infection numbers firstly became zero.

In order to apply our model, we need to estimate the parameters. The median incubation period of COVID-19 is approximately 5 days (Lauer et al., 2020) and the average duration of incubation is 1/*σ* according to our model, thus we set *σ* = 1/5. Averaging the values from 24 January 2020 to 16 February 2020 in Fig. 1, we obtain the recovery rate *α*(*t*) and the disease-induced death rate *γ*(*t*) for *t* ∈ [*t*_0_, *t*_4_). At time *t*_4_, the rates switch to the values averaged from 17 February 2020 to 31 March 2020.

The basic reproduction number *R*_0_ represents the average number of people that one person with SARS-CoV-2 is likely to infect in an otherwise susceptible population. In our model, it is straightforward that

(6) $$R_{0}=\frac{\beta_{20}S_{0}}{\alpha +\gamma }+\frac{\beta_{30}S_{0}}{\sigma },$$

where *S*_0_ = *N* is the total population in Wuhan. The basic reproduction number is of great importance in measuring the risk of transmission, and its estimation ranges from 2.2 to 5.7 (Imai et al., 2020; Li et al., 2020; Read et al., 2020; Sanche et al., 2020; Tuite & Fisman, 2020; Wu et al., 2020). Moreover, the percentage of transmission occurring prior to symptom onset is 33.7*%* in Wuhan (Casey et al., 2020), and this proportion can be approximated by the ratio $\frac{\beta_{30}N}{\sigma }/R_{0}$. There are around 11, 081, 000 inhabitants in Wuhan (Chang Jiang Ri Bao, 2019), hence *N* = 11, 081, 000. The recovery rate *α* = 0.055359 and disease-induced death rate *γ* = 0.0049855 are the average value from 24 January 2020 to 31 March 2020. Taking *R*_0_ = 2.6, we have *β*_20_ = 9.38741 × 10^−9^ and *β*_30_ = 1.58145 × 10^−8^.

Studies have shown that the novel coronavirus can survive for up to 72 h on plastic and stainless steel, less than 24 h on cardboard and less than 4 h on copper(Van Doremalen et al., 2020). Within the range, we choose *d* = 1 meaning that the average life span of SARS-CoV-2 in the environment is one day. Since there is no data about the SARS-CoV-2 concentration in Huanan seafood wholesale market, we use the number of confirmed cases from 8 December 2019 to 13 January 2020 given by Fig. 1 in (Li et al., 2020) to estimate corresponding parameters. Set *S*(*t*_0_) = 11, 081, 000 and the choice of initial time indicates *I*(*t*_0_) = 1. Let *E*(*t*_0_) = 2 since two confirmed cases developed symptoms in the next five days. Assuming that *p*_10_ = 0.00015, the *V*(*t*)-to-*S*(*t*) transmission rate and the discharging rate of the novel coronavirus are estimated to be *β*_10_ = 2.32967 × 10^−7^ and *μ* = 5084 by using the nonlinear least-squares solver *lsqnonlin* built in MATLAB.

Continuing with the above estimated parameter values, we use the data reported by Wuhan Municipal Health Commission from 20 January 2020 to 16 February 2020 to estimate *m*_1_(*t*) and *m*_2_(*t*) for *t* ∈ [*t*_2_, *t*_4_) and *m*_3_(*t*) for *t* ∈ [*t*_3_, *t*_4_). Then we obtain the estimated value of *m*_4_(*t*) and *m*_5_(*t*) for *t*⩾*t*_4_ by fitting the reported number of confirmed cases from 16 February 2020 to 31 March 2020. All the parameter values are summarized in Table 2.

**Table 2:**
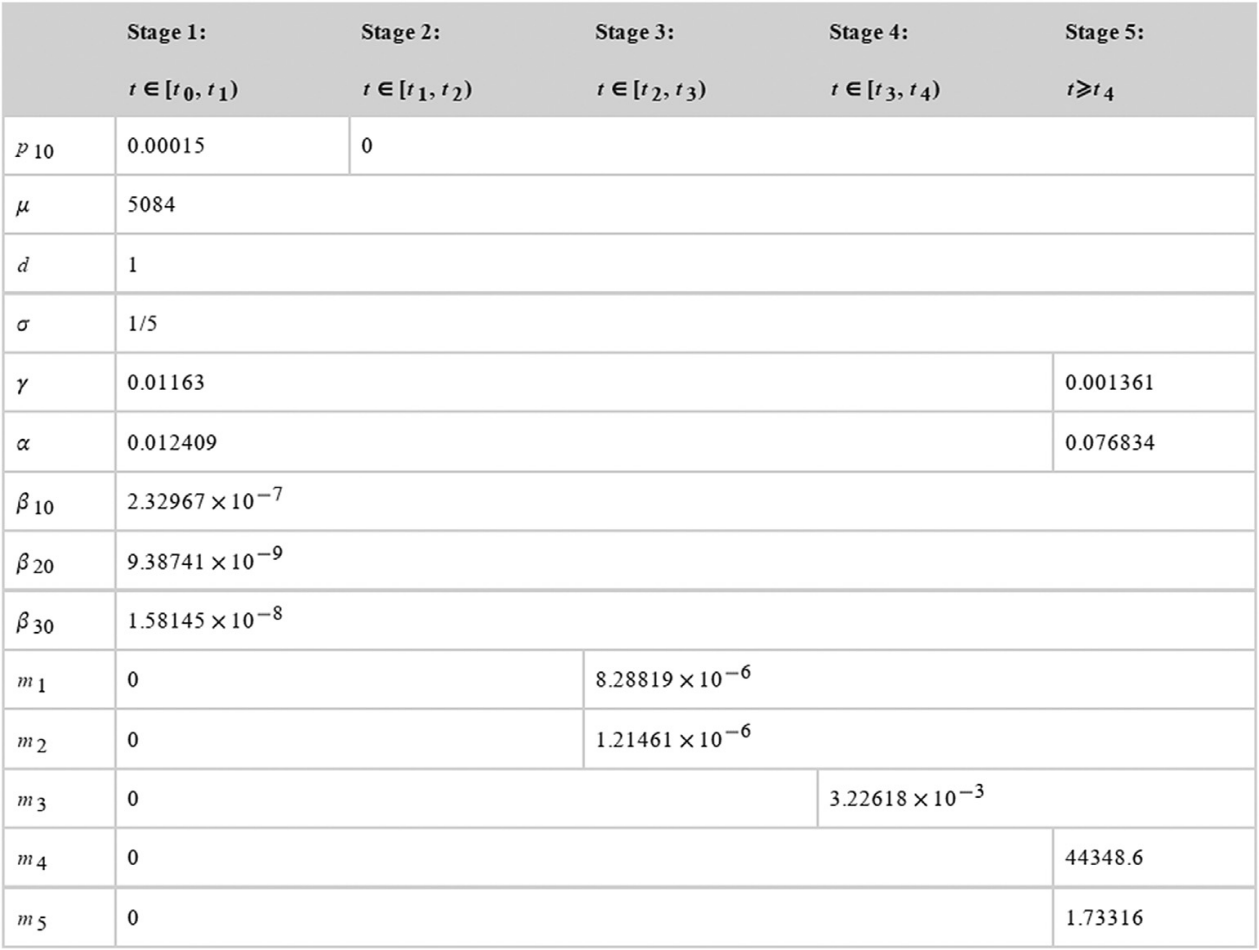
Parameter estimates for the COVID-19 outbreak in Wuhan.

With the parameter values in Tables 1 and 2, the numerical simulation of model (5) is shown in Fig. 2, in comparison with the reported data. One should realize that the confirmed case number is much lower than the real infected population in the beginning since most of the patients were not identified until early February. By 31 January 2020, there were 32, 655 individuals infected by the novel coronavirus in mainland China but only 11, 791 of them were reported (The Novel Coronavirus Pne, 2020). The situation could not be better in Wuhan. The reported number of active infections drops to zero on 26 April 2020 and we ignore the confirmed cases after then since imported infections become a major source. But our model takes a much longer time for the disease to be eliminated. This is because once we have fixed per capita recovery rate to a constant, the decreasing rate becomes very small when *I*(*t*) approaches to zero, while the real recovery rate gets relatively high when active cases drop to a low level as is shown in Fig. 1. There are 50, 333 individuals in total infected by the novel coronavirus by 26 April 2020 and the active infection number peaks on 18 February 2020 being 38, 020. In our simulation *I*(*t*) reaches its maximum 38, 577 on 18 February 2020 (*t* = 73). By 26 April 2020, the accumulative infection number is 74, 075. The outbreak ends on 12 July 2020 (*t* = 218) (in the sense that *I*(*t*) firstly decreases to 1) with the epidemic final size 74, 076.

## 3 Exploration of non-pharmaceutical interventions

We have seen in the preceding section that the disease dynamics described by the model (5) with the parameters given in Tables 1 and 2 fits very well with the actual outbreak of COVID-19 in Wuhan. In this section, we numerically explore the impact of interventions by varying the values of some parameters that present these control measures.

**Fig. 3:**
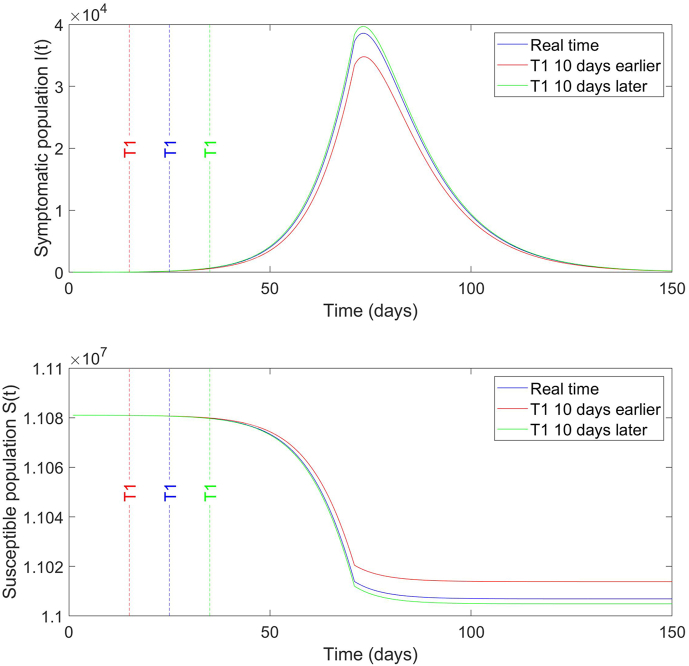
The disease dynamics with different market closure timing *t*_1_.

**Fig. 4:**
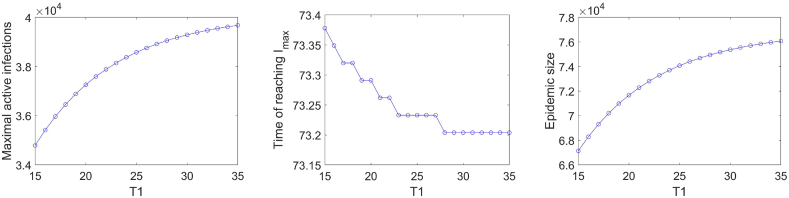
The impact of timing *t*_1_ of the closure of Huanan seafood wholesale market — left: on maximal number of active infections; central: on the time reaching the maximal infections; right: on the epidemic size.

We first look at the impact of the closure of Huanan seafood wholesale market. If it was not closed, a scenario that the time point *t*_1_ is no longer available and hence *p*_1_(*t*) = *p*_10_ = 0.00015 for all *t*⩾*t*_0_, numerical simulation of the model (5) then shows that the number of *I*(*t*) at peak would be higher, reaching 40, 157 on 18 February 2020 (*t* = 73), and the outbreak would never end since environment-to-human transmission would have persisted. If the market was closed but on different dates, the impact is shown by the numerical results in Fig. 3, Fig. 4.

**Fig. 5:**
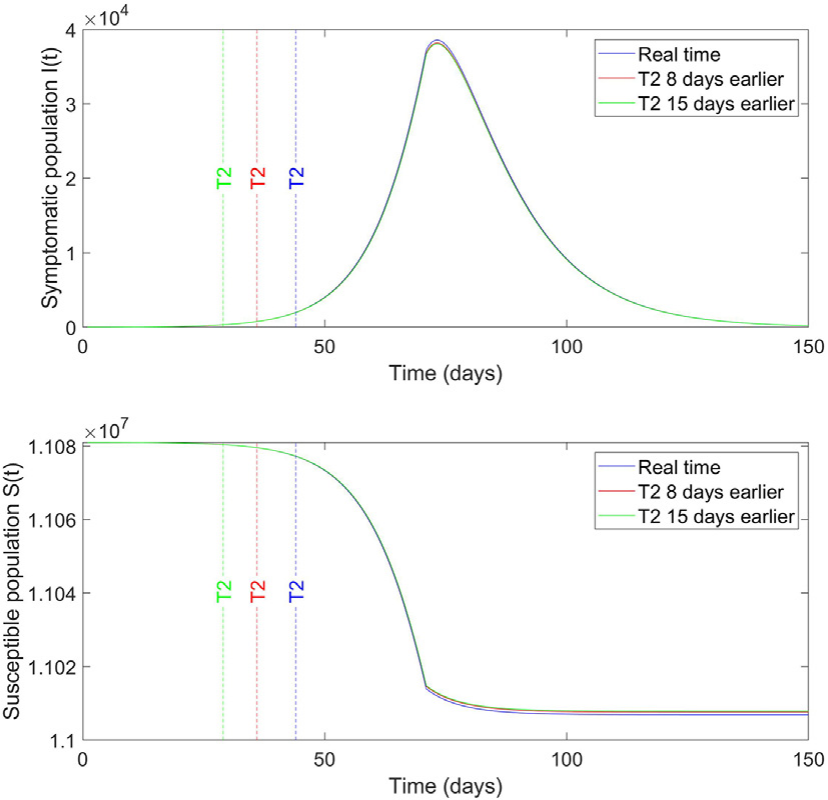
The disease dynamics with different timing *t*_2_ of human-to-human transmission announcement.

**Fig. 6:**
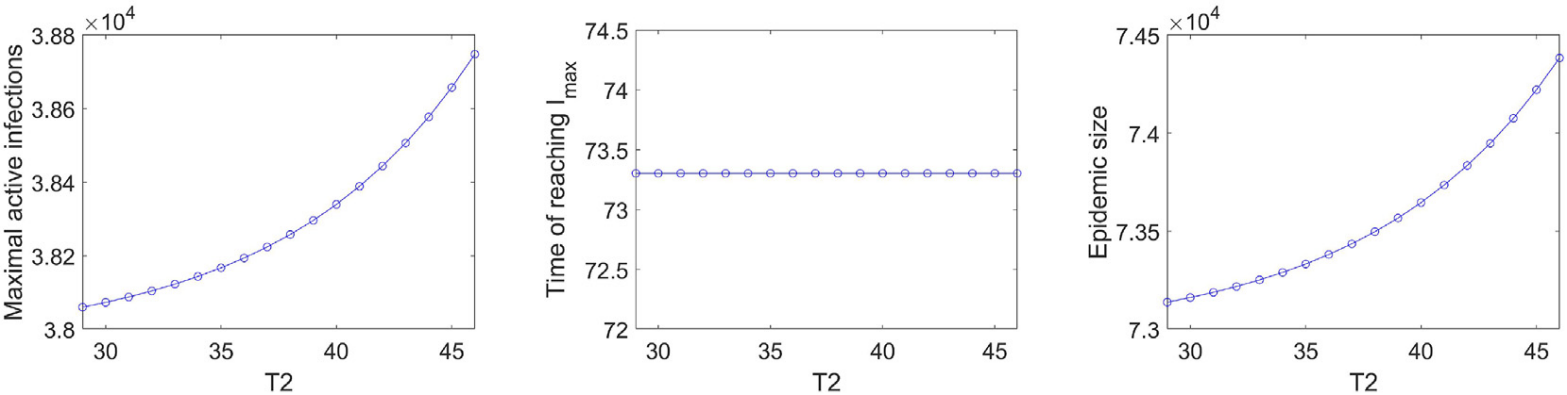
The impact of timing *t*_2_ of making the public known that SARS-CoV-2 can be transmitted from human to human — left: on maximal number of active infections; middle: on the time reaching the maximal infections; right: on the epidemic size.

Next we investigate the impact of media coverage. Media coverage affects the dynamics of epidemic via the change of individuals' behaviours, reflected by *m*_1_(*t*) and *m*_2_(*t*) in our model. If *m*_1_(*t*) = *m*_2_(*t*) = 0 for all *t*⩾*t*_0_, the maximal number of active infections would increase to 54, 604 reached on 20 February 2020 (*t* = 75), and the total epidemic size would increase to 111, 026 with the disease outbreak ending on 19 July 2020 (*t* = 225). With *m*_1_(*t*) and *m*_2_(*t*) switching on at *t*_2_, media coverage starts affecting human's behaviours, making less susceptible people available for infection. We can also explore the impact of altering *timing* *t*_2_, which is illustrated in Fig. 5, Fig. 6 by the numerical results for the model.

**Fig. 7:**
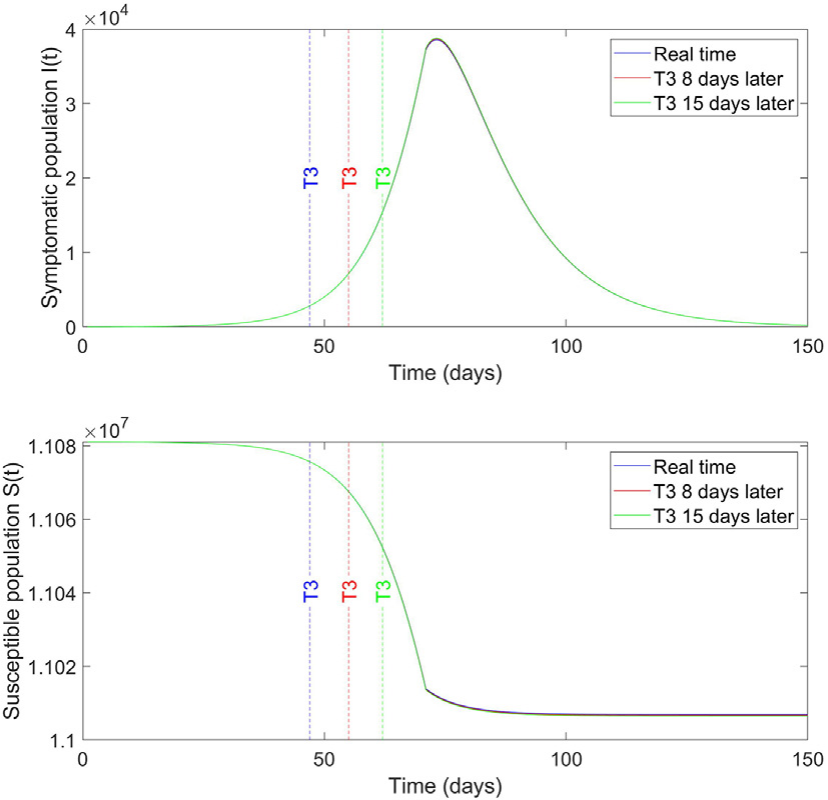
The disease dynamics with different lockdown timing *t*_3_.

**Fig. 8:**
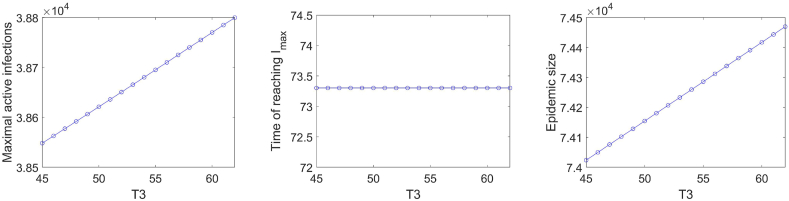
The impact of altering timing *t*_3_ of lockdown in Wuhan city — left: on maximal number of active infections; central: on the time reaching the maximal infections; right: on the epidemic size.

Next, the impact of lockdown. Assuming that there was no lockdown since *t* = *t*_3_, then *m*_3_(*t*) = 0 for all *t*⩾*t*_0_. Numerical simulation of our model indicates that the disease outbreak would peak on 18 February 2020 (*t* = 73) with 38, 911 active infections and would end on 12 July 2020 (*t* = 218) with the total epidemic size being 74, 730 cases. We can also examine the impact of the lockdown timing, as demonstrated by the numerical simulations of our model, presented in Fig. 7, Fig. 8, when *t*_3_ varies alone between *t*_2_ and *t*_4_.

**Fig. 9:**
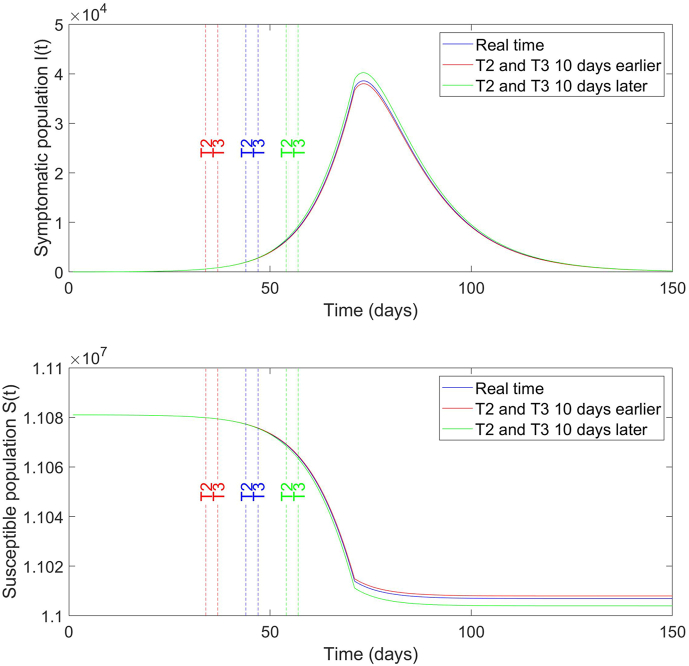
The impact of synchronously altering *t*_2_ and *t*_3_ in the sense of *t*_3_ = *t*_2_ + 3 on the disease dynamics.

**Fig. 10:**
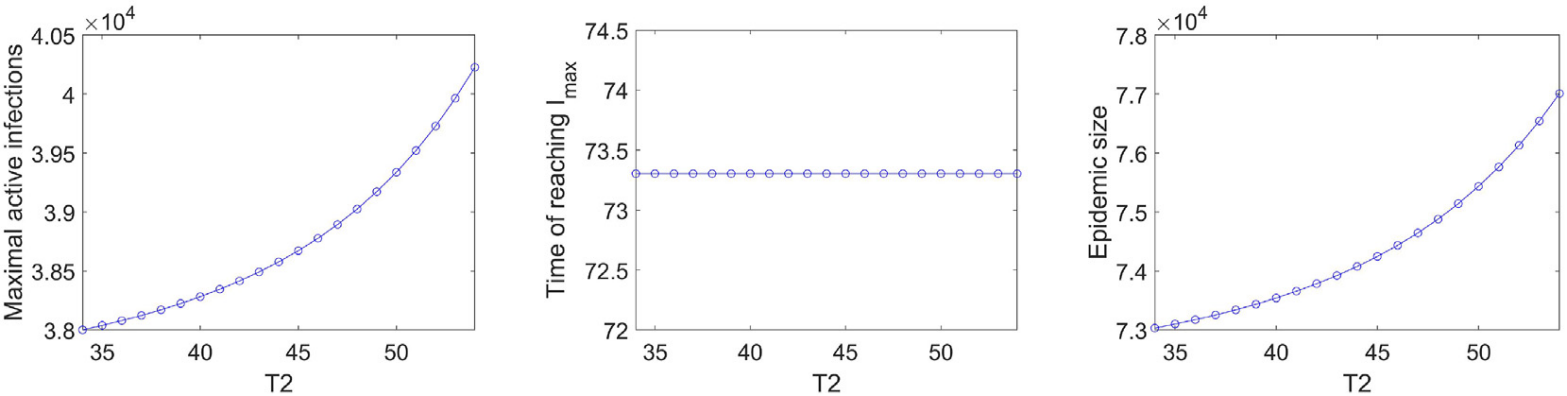
The impact of synchronously altering *t*_2_ and *t*_3_ in the sense of *t*_3_ = *t*_2_ + 3 — left: on maximal number of active infections; central: on the time reaching the maximal infections; right: on the epidemic size.

We may also look at the consequence of altering the pair of two time points *t*_2_ and *t*_3_. For simplicity, we let *t*_2_ and *t*_3_ vary synchronously in the sense of *t*_3_ = *t*_2_ + 3, which approximately accounts for a scenario that three days are needed to prepare for a lockdown after realizing the severity of the disease due to massive human-to-human transmission. Fig. 9 presents the corresponding disease dynamics with respect to the time variable, while Fig. 10 shows how such a change in this pair of time critical points affects the maximal number of active infections, the time reaching the maximal infections, and the epidemic size.

As mentioned in the introduction, the addition of extra medical resources played a crucial and even decisive role in controlling the epidemic in Wuhan. Using our model with the estimated model parameters, we can also investigate how the timings of obtaining these resources affect the disease dynamics. If no such additional resources were ever available at all, then *t*_4_ = *∞*. The outbreak would have lasted for more than ten years, affecting the majority of population, and the maximal number of active infections would reach 421, 531 on 7 July 2020 (*t* = 213). To some extent, this result explains why this novel pneumonia threatens public health and interventions are essential.

**Fig. 11:**
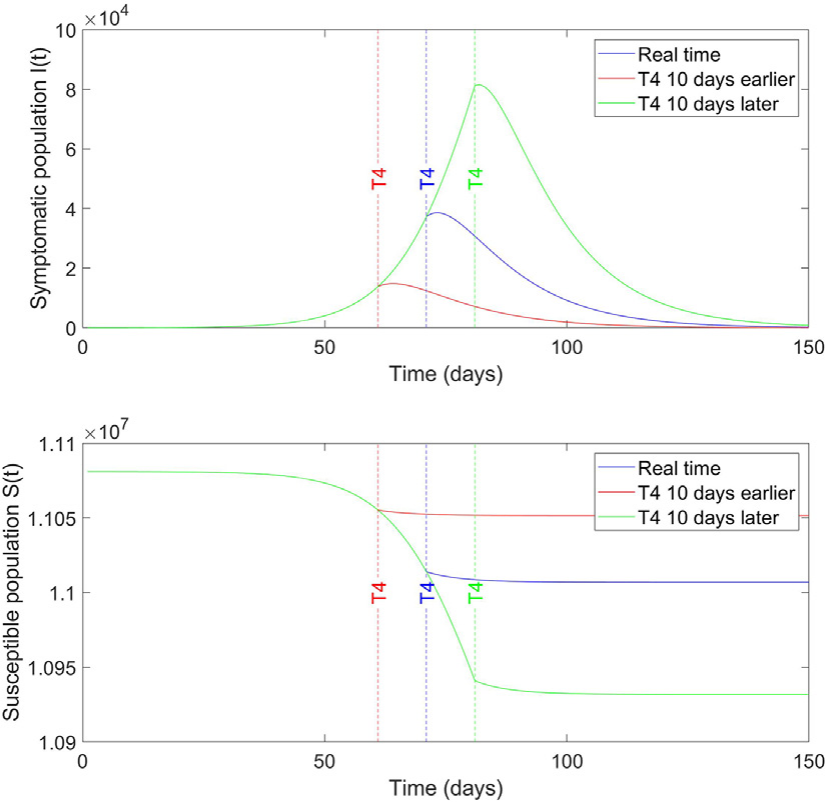
The disease dynamics with different timing *t*_4_ of providing extra medical support to Wuhan.

**Fig. 12:**
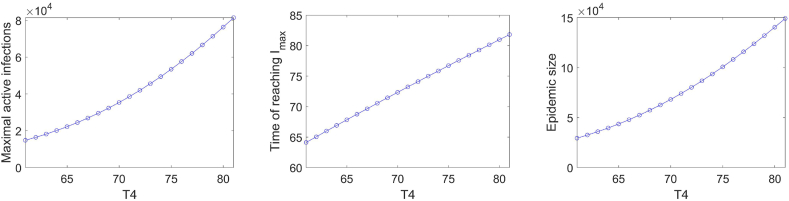
The impact of timing *t*_4_ of providing extra medical support — left: on maximal number of active infections; central: on the time reaching the maximal infections; right: on the epidemic size.

Notice that after *t*_4_, *m*_4_(*t*) is switched on to 44348.6 which is significantly larger than *m*_5_(*t*), leading to 1/(1 + *m*_4_) = 0.00002255 and 1/(1 + *m*_5_) = 0.36587686. This means that after *t*_4_, only a very tiny proportion of *S* group people (mainly frontline health workers) have chances of contacting *I* group individuals, while there is still a relative large fraction of *S* group people who have chances to contact *E* group individuals. This well supports the role (purpose) of *m*_4_(*t*) and *m*_5_(*t*), as explained in the introduction. Thus, variation in timing *t*_4_ makes significant differences on the disease dynamics. Corresponding numerical results are shown in Fig. 11, Fig. 12.

## 4 Conclusion and discussion

We have proposed a mathematical model in the form of ordinary differential equations to re-examine the COVID-19 epidemics in Wuhan City in China where this disease initially broke out in late 2019 and early 2020. The model is a modification of the classic Kermack-McKendrick SIR model by incorporating four major control measures (see (M1)-(M4) in the introduction), implemented in Wuhan City during the epidemic in that city. The focus is to examine the effect of those NPIs. Realizing that those control measures essentially reduced the number of susceptible individuals that are *available for infection*, we have introduced a new notion called “available susceptible population” which is further differentiated to “*I*-available susceptible” and “*E*-available susceptible” subgroups, characterized by two fraction functions *p*_2_(*t*) and *p*_3_(*t*). These two fraction functions capture the main features of measures (M2), (M3) and (M4), and have been given the particular forms in (4). We have used the values of some model parameters from published research results, and estimated the other model parameters by using the data publicly available for COVID-19 epidemics in Wuhan and the nonlinear least-squares solver *lsqnonlin* built in MATLAB. With these values of model parameters, we have numerically explored the impact of those NPIs. particularly the impact of the timings of these interventions, on the epidemics. These results can help us review the responses to the COVID-19 outbreak in Wuhan and seek improvement in handling similar disease outbreaks. For example, our numerical simulation results indicate that the impact of *t*_2_ and *t*_3_ is not as significant as that of *t*_1_ and *t*_4_. This seems to suggest that cutting off the environment-to-human transmission and increasing the capability of identifying and isolating infected people are more effective than media coverage and lockdown. Moreover, the proposed model can also offer a framework for studying disease dynamics of COVID-19 and/or other similar diseases in other places, accordingly helping us better prepare for possible future outbreak of similar diseases.

We point out that our results of model simulation may not reflect the exact situation of the real world. This is because the parameters are estimated by fitting the reported data, which in general were subject to the capability of testing and various other restrictions. Different datum sets may lead to dramatically different results. As such, obtaining reliable data should be crucial for making reliable prediction by the model. In this regards, we would like to mention the work (Huo et al., 2021) (thank Dr Shigui Ruan for sharing this most recent work with us) which indeed focuses on estimating *asymptotical and undetected cases* (not reflected in the dada of reported cases), as well as the total cases for the COVID-19 outbreak in Wuhan. For this purpose, the compartmental model in (Huo et al., 2021) contains three extra compartments: *A*(*t*) — the number of individuals that are infectious but asymptomatic at time *t*, *D*(*t*)— the number of cases that were detectable but not yet detected or reported at time *t*, and *W*(*t*)— the number of cases that has been reported at time *t*. Another feature of the model in (Huo et al., 2021) is that the infectious and symptomatic class is further divided into detectable and undetectable classes to account for the situation in Wuhan when there is a huge lack of efficient and reliable tests when COVID -19 initially broke out there. With these missing pieces putting into the model, the results of (Huo et al., 2021) provide some very interesting and useful estimations on the actual infection cases in Wuhan. Note that the model in (Huo et al., 2021) and our model (2) do share an important common nature: some model parameters are piecewise time dependent and the breaking down of the time interval reflects the timings of implementations of some major NPIs.

We remark that as far as parameter estimations for ODE and PDE models for infectious diseases are concerned, there is a large literature on various methods. For example, the recent work (Demongeot et al., 2020) and (Liu et al., 2020) used a more model specific method to estimate some model parameters in some ODE models. In (Huo et al., 2021), the authors used the Monte Carlo Marckov Chain method built in the software Stan. Here we used the nonlinear least-squares solver *lsqnonlin* built in MATLAB to estimate some parameters. Since the methods of parameter estimation and data fitting are not the focus of this work, we avoid a further review and discussion in this paper.

We also note that in (4), the first part on the right hand side of the formulas for *p*_2_ and *p*_3_, 1/[1 + *q*(*t*)] with $q\left(t\right)=m_{1}\left(t\right)I\left(t\right)+m_{2}\left(t\right)\tilde{\Delta }I\left(t\right)$ accounting for a kind of “weighted information of infections” serves as a decreasing factor. For this purpose, instead of 1/[1 + *q*(*t*)], other types of decreasing functions can also be used. One example is the exponential decay function *e*^−*q*(*t*)^. Similarly, the other parts in (4) as decreasing functions of *m*_*i*_(*t*) (*i* = 3, 4, 5) can also have other options in their forms. Choosing the most suitable form for each of them remains an interesting and open problem.

Notice that we have divided this outbreak into five stages for mathematical simplification. The choice of *t*_4_, the supposed date when extra medical resources were available, is only for such purpose. In fact, the construction of hospitals and recruitment of medical staff began in the end of January and lasted for a couple of weeks. The disease-induced death rate and recovery rate varies day by day. In this model, we have simply set the critical time point *t*_4_ and switch the values of corresponding parameters.

We have included the concentration of SARS-CoV-2 in the environment into the model. Since most early cases were believed to be infected by the viruses discharging from wildlife traded in Huanan seafood wholesale market, we have assumed that no more people catch COVID-19 by exposure to coronaviruses in the environment after closure of the market. However, this is a bit too simple and ideal, as some studies have shown that the novel coronavirus can exist several hours in the air and survive up to a couple of days on surfaces. An improvement should include this infection mechanism for all *t*⩾*t*_0_ and the effect of disinfection of the environment, particularly those key places that provide essential services and have substantially large chances of being visited by infected individuals, such as, hospitals, supermarkets, convenient stores, gas stations, public transportations, *etc.*

## Declaration of competing interest

The authors declare that they have no known competing financial interests or personal relationships that could have appeared to influence the work reported in this paper.

The authors declare the following financial interests/personal relationships which may be considered as potential competing interests:

## References

[bib1] Casey M., Griffin J., McAloon C.G. (2020). Estimating pre-symptomatic transmission of COVID-19: A secondary analysis using published data.

[bib2] Chang Jiang Ri Bao (Changjiang Daily) (March 25, 2019). Statistical communique of national economic and social development of Wuhan in 2018. http://cjrb.cjn.cn/images/2019-03/25/6/25R06-07C_Print.pdf.

[bib3] Chang X., Liu M., Jin Z. (2020). Studying on the impact of media coverage on the spread of COVID-19 in Hubei Province, China. Mathematical Biosciences and Engineering.

[bib4] Chen Y., Guo D. (2016). Molecular mechanisms of coronavirus RNA capping and methylation. Virologica Sinica.

[bib5] Chen Y., Liu Q., Guo D. (2020). Emerging coronaviruses: Genome structure, replication, and pathogenesis. Journal of Medical Virology.

[bib6] Cui Q., Hu Z., Li Y. (2020). Dynamic variations of the COVID-19 disease at different quarantinestrategies in Wuhan and mainland China. Journal of Infection and Public Health.

[bib7] Demongeot J., Griette Q., Magal P. (2020). SI epidemic model applied to COVID-19 data in mainland China. R. Soc. Open Sci..

[bib8] Funk S., Gilad E., Watkins C. (2009). The spread of awareness and its impact on epidemic outbreaks. Proceedings of the National Academy of Sciences.

[bib9] Ge X.Y., Li J.L., Yang X.L. (2013). Isolation and characterization of a bat SARS-like coronavirus that uses the ACE2 receptor. Nature.

[bib10] Hellewell J., Abbott S., Gimma A. (2020). Feasibility of controlling COVID-19 outbreaks by isolation of cases and contacts. The Lancet Global Health.

[bib11] Huang C., Wang Y., Li X. (2020). Clinical features of patients infected with 2019 novel coronavirus in Wuhan, China. The Lancet.

[bib12] Hui D.S.C., Zumla A. (2019). Severe acute respiratory syndrome: Historical, epidemiologic, and clinical features. Infectious Disease Clinics of North America.

[bib13] Huo X., Chen J., Ruan S. (2021). Estimating asymptotic, undetected and total cases for the COVID-19 outbreak in Wuhan: A mathematical modeling study. BMC Infectious Diseases.

[bib14] Imai N., Cori A., Dorigatti I. (2020). Report 3: Transmissibility of 2019nCoV.

[bib15] Kahn J.S., McIntosh K. (2005). History and recent advances in coronavirus discovery. The Pediatric Infectious Disease Journal.

[bib16] Lai S., Ruktanonchai N.W., Zhou L. (2020). Effect of non-pharmaceutical interventions to contain COVID-19 in China. Nature.

[bib17] Lauer S.A., Grantz K.H., Bi Q. (2020). The incubation period of coronavirus disease 2019 (COVID-19) from publicly reported confirmed cases: Estimation and application. Annals of Internal Medicine.

[bib18] Li Q., Guan X., Wu P. (2020). Early transmission dynamics in Wuhan, China, of novel coronavirus-infected pneumonia. New England Journal of Medicine.

[bib19] Liu N., Chen Z., Bao G. (2021). Role of media coverage in mitigating COVID-19 transmission: Evidence from China. Technological Forecasting and Social Change.

[bib20] Liu Z., Magal P., Seydi O., Webb G. (2020). Understanding unreported cases in the 2019-nCov epidemic outbreak in Wuhan, China, and the importance of major public health interventions. MPDI Biol.

[bib21] Read J.M., Bridgen J.R.E., Cummings D.A.T. (2020). Novel coronavirus 2019-nCoV: Early estimation of epidemiological parameters and epidemic predictions.

[bib22] Sanche S., Lin Y.T., Xu C. (2020). High contagiousness and rapid spread of Severe acute respiratory syndrome coronavirus 2. Emerging Infectious Diseases.

[bib23] Shen M., Peng Z., Guo Y. (2020). Lockdown may partially halt the spread of 2019 novel coronavirus in Hubei province, China. International Journal of Infectious Diseases.

[bib24] Tang B., Bragazzi N.L., Li Q. (2020). An updated estimation of the risk of transmission of the novel coronavirus (2019-nCov). Infectious Disease Modelling.

[bib25] Tan W., Zhao X., Ma X. (2020). A novel coronavirus genome identified in a cluster of pneumonia cases – Wuhan, China 2019-2020. China CDC Weekly.

[bib26] The Novel Coronavirus Pneumonia Emergency Response Epidemiology Team (2020). The epidemiological characteristics of an outbreak of 2019 novel coronavirus diseases (COVID-19) in China. Chinese Journal of Epidemiology.

[bib27] Tuite A.R., Fisman D.N. (2020). Reporting, epidemic growth, and reproduction numbers for the 2019 novel coronavirus (2019-nCoV) epidemic. Annals of Internal Medicine.

[bib28] Tyrrell D.A., Almeida J.D., Cunningham C.H. (1975). Coronaviridae. Intervirology.

[bib29] Van Doremalen N., Bushmaker T., Morris D.H. (2020). Aerosol and surface stability of SARS-CoV-2 as compared with SARS-CoV-1. New England Journal of Medicine.

[bib30] Wang L.F., Shi Z., Zhang S. (2006). Review of bats and SARS. Emerging Infectious Diseases.

[bib31] World Health Organization (WHO): Middle East respiratory syndrome coronavirus (MERS-CoV). https://www.who.int/emergencies/mers-cov/en (accessed December 4, 2020).

[bib32] World Health Organization (WHO): Naming the coronavirus disease (COVID-19) and the virus that causes it. https://www.who.int/emergencies/diseases/novel-coronavirus-2019/technical-guidance/naming-the-coronavirus-disease-(covid-2019)-and-the-virus-that-causes-it (accessed December 4, 2020).

[bib33] World Health Organization (WHO): Severe Acute Respiratory Syndrome (SARS). https://www.who.int/ith/diseases/sars/en/(accessed December 4, 2020).

[bib34] World Health Organization (WHO): WHO coronavirus disease (COVID-19) dashboard. https://covid19.who.int/(accessed December 9, 2020).

[bib35] Wuhan Municipal Health Commission: http://wjw.wuhan.gov.cn/ztzl_28/fk/yqtb/(accessed May 26, 2020).

[bib36] Wu J.T., Leung K., Leung G.M. (2020). Nowcasting and forecasting the potential domestic and international spread of the 2019nCoV outbreak originating in Wuhan, China: A modelling study. The Lancet.

[bib37] Yang Z., Zeng Z., Wang K. (2020). Modified SEIR and AI prediction of the epidemics trend of COVID-19 in China under public health interventions. Journal of Thoracic Disease.

[bib38] Zhou W., Wang A., Xia F. (2020). Effects of media reporting on mitigating spread of COVID-19 in the early phase of the outbreak. Mathematical Biosciences and Engineering.

[bib39] Zumla A., Hui D.S., Perlman S. (2015). Middle East respiratory syndrome. The Lancet.

